# Oncotherapeutic Protein Kinase Inhibitors Associated With Pro-Arrhythmic Liability

**DOI:** 10.1016/j.jaccao.2021.01.009

**Published:** 2021-03-16

**Authors:** Johan Z. Ye, Finn B. Hansen, Robert W. Mills, Alicia Lundby

**Affiliations:** a Department of Biomedical Sciences, Faculty of Health and Medical Sciences, University of Copenhagen, Copenhagen, Denmark; b Department of Health Technology, Section for Bioinformatics, Technical University of Denmark, Lyngby, Denmark; c The Novo Nordisk Foundation Center for Protein Research, Faculty of Health and Medical Sciences, University of Copenhagen, Copenhagen, Denmark

**Keywords:** alectinib, cardiac arrhythmia, cardiotoxicity, crizotinib, ibrutinib, idelalisib, nilotinib, osimertinib, pharmacovigilance, ponatinib, protein kinase inhibitor, ribociclib, risk models, trametinib U.S. Food and Drug Administration Adverse Event Reporting System, AF, atrial fibrillation, HLGT, high level group terms, HLT, high level terms, FAERS, FDA Adverse Event Reporting System, FDA, U.S. Food and Drug Administration, MedDRA, Medical Dictionary for Regulatory Activities, PKI, protein kinase inhibitor, PT, preferred term, ROR, reporting odds ratio, SOC, system organ classes

## Abstract

**Background:**

Ibrutinib is a protein kinase inhibitor that has been widely successful in treating multiple common variations of B-cell cancers. However, an unfortunate side effect of ibrutinib is that it predisposes patients to development of atrial fibrillation.

**Objectives:**

The purpose of this study was to assess other commonly prescribed protein kinase inhibitors for similar pro-arrhythmic liability.

**Methods:**

This study comprehensively evaluated data from the U.S. Food and Drug Administration adverse events reporting system and determined the reporting of cardiac arrhythmia attributed to kinase inhibitor therapy using a multivariable logistic regression model. We evaluated 3,663,300 case reports containing 23,067 cases of atrial fibrillation and 66,262 cases of cardiac arrhythmia. In total, 32 protein kinase inhibitors were evaluated, almost all of which are oncotherapeutics.

**Results:**

Seven protein kinase inhibitors were associated with a significant increase in the odds of atrial fibrillation (ibrutinib, ponatinib, nilotinib, ribociclib, trametinib, osimertinib, and idelalisib). Assessment of broader pro-arrhythmic toxicity suggested a ventricular-specific liability for nilotinib and a bradyarrhythmia risk with alectinib and crizotinib.

**Conclusions:**

Compounds that result in the inhibition of a number of protein kinases are associated with an increased risk of cardiac rhythm disturbances. The mechanisms driving the arrhythmogenic effects remain to be discovered, but this study presents an important step in identifying and prioritizing the study of these protein kinase signaling pathways.

Inhibition of protein kinases has emerged as a successful therapeutic strategy in the treatment of several cancers. Despite their success as oncotherapeutics, some protein kinase inhibitors (PKIs) have significant cardiac liabilities, which have led to the emergence of specialized cardio-oncologic studies and patient care (1,2). Among the successful therapeutics is ibrutinib, which is used to treat chronic lymphocytic leukemia, the most common form of leukemia in adults. However, this tyrosine kinase inhibitor has been associated with increased risk of several cardiovascular toxicities, and a meta-analysis of 20 studies found the pooled rate of atrial fibrillation (AF) for patients treated with ibrutinib to be 3.3 per 100 person-years (range, 2.5. to 4.1), compared to 0.84 per 100 person-years (0.32 to 1.6) for non-ibrutinib therapy (3, 4, 5, 6). The therapeutic target of ibrutinib is Bruton’s tyrosine kinase (*BTK*), which is irreversibly inhibited by the drug. Bruton’s tyrosine kinase is essential for activation of several pathways necessary for chronic lymphocytic leukemia cell survival, including the Akt, ERK, and NF-κB pathways (7). Ibrutinib also inhibits a number of other tyrosine kinases, albeit with lower affinities. A recent study singled-out the off-target inhibition of the tyrosine-protein kinase CSK as a mechanism by which Ibrutinib can cause AF (8).

To assess the extent of proarrhythmic liability within the pharmaceutical class of PKIs, we investigated whether other PKIs exhibit similar adverse effects as ibrutinib. We evaluated the association between PKIs and the reporting of AF by analysis of the medication adverse events database maintained by the U.S. Food and Drug Administration (FDA). In addition, we evaluated additional electrophysiological perturbations associated with these compounds beyond AF by first evaluating these compounds for associations with cardiac arrhythmias in general followed by a post hoc analysis of the arrhythmia etiologies underlying the signals. Most pharmacovigilance studies have used disproportionality analysis similar to Chi-squared test to quantify the association between drug and adverse event (5). Here, we make use of a standard probabilistic machine learning algorithm, logistic regression, to quantify the propensity to cardiac rhythm disorders as a side effect of protein kinase inhibition, while also accounting for confounding effects. Investigation of shared targeted kinases among PKIs with proarrhythmic liability could potentially improve our understanding of the role of specific kinases in cardiac rhythm maintenance, and hence the detrimental impact on cardiac rhythm by kinase inhibition. The associations presented herein lay the groundwork for determining which subset of on- and off-target kinome modifications drive the proarrhythmic cardiotoxicity within this class of compounds with the goal of minimizing these effects in future drug development efforts.

## Methods

### Data sources

The FDA Adverse Event Reporting System (FAERS) is publicly available and anonymized; therefore, this analysis did not require ethics committee approval. Full details on data sourcing and vetting are in the Supplemental Appendix. The Protein Kinase Inhibitor Database (PKIDB) was assessed for information on PKIs used clinically or in clinical trials (9). To analyze for proarrhythmic effects of protein kinase inhibition, reports from publicly available adverse event cases from the FAERS were collated and assessed (10). Data in FAERS from the 5-year period: Q3 of 2014 through Q2 of 2019 were examined. The reports were rigorously vetted, and the final analyses contained 3,663,300 reports. The Medical Dictionary for Regulatory Activities (MedDRA) for standardized descriptor terminology and classification was used to organize items into 5 different hierarchical groups: 1) system organ classes (SOCs); 2) high level group terms (HLGTs); 3) high level terms (HLTs); 4) preferred terms (PTs); and 5) lowest level terms.

### Signal detection algorithms

Association of PKIs with AF or other cardiac arrhythmias was assessed using established signal detection algorithms which calculate surrogate measures of the association between treatment and effect for statistical testing. Two types of drug-effect signal detection methods are typically used in pharmacovigilance studies, those based on disproportionality analysis and those based on multivariable modeling (11). The disproportionality analysis methods use a univariable approach that focuses on the relative proportions of patients experiencing the adverse event. This analysis is predominantly based on 2-by-2 contingency tables, evaluating the effect of treatment from summary statistics allowing for analysis without using patient-level information. This approach is simple and computationally efficient but focuses solely on differences in proportions and cannot account for possible confounding effects in a data set. In contrast, multivariable models analyze multiple variables in parallel, with logistic regression being the most widely used. Logistic regression is a machine learning algorithm, which is preferable due to its ability to quantify variable effects without any assumptions of the underlying data distribution. The regression first applies a nonlinear transformation followed by a 2-class regression. Because the model is flexible and uses a probabilistic approach, it has multiple usages. In this case, the relative effect of variables (treatment with a specific PKI) to separate patients experiencing the specific adverse event from the other reports was quantified. The primary advantage of this method is the ability to account for confounding variables associated with patient characteristics (e.g., age, sex, and comorbidities). A need to account for confounding variables dictated the use of a multivariable model over a disproportionality analysis in this manuscript. However, this requires that patient-level data are well-curated.

### Statistics

The association of PKIs with AF or cardiac arrhythmia is presented as odds ratios (ORs) of reported new-onset disease attributed to PKI use compared to reported new-onset disease within the reference population. Specifically, the odds quantify the relative probability that a reported adverse event is a cardiac arrhythmia (or AF specifically) compared to the probability that it is any other kind of adverse event. The odds are calculated within a specified population, and the odds ratio compares these odds to the odds within a reference population. The reference population consists of the pooled reports for all other non-PKI drugs reported on within the FAERS database and vetted as described above. In the present analysis, each PKI is independently assessed against the reference population after controlling all reports for their internal composition of confounding factors. For each PKI, the reporting odds ratio (ROR) with 95% confidence intervals (CIs) are shown, and the ROR is taken as being significantly increased at the α = 0.05 level adjusted for multiple comparisons using the strict Bonferroni correction. Statistical power assessment finds that in this analysis, a drug with 500 reports (minimum reports cutoff) could be detected at an OR of at least 2.47 with a power of 80%, whereas a drug with 4,000 reports (approximate median number of reports for analyzed PKIs) can be detected at an OR of at least 1.47 with a power of 80%. All analyses were conducted in Python 3.7.6, using a combination of Statsmodel 0.11.0, Numpy 1.18.1, Pandas 1.0.1, Scikit-learn 0.22.1, Matplotlib 3.1.3, and Seaborn 0.10.0 (12, 13, 14, 15, 16).

## Results

### Age, sex, and comorbidities identified as confounding effects

**Figure 1:**
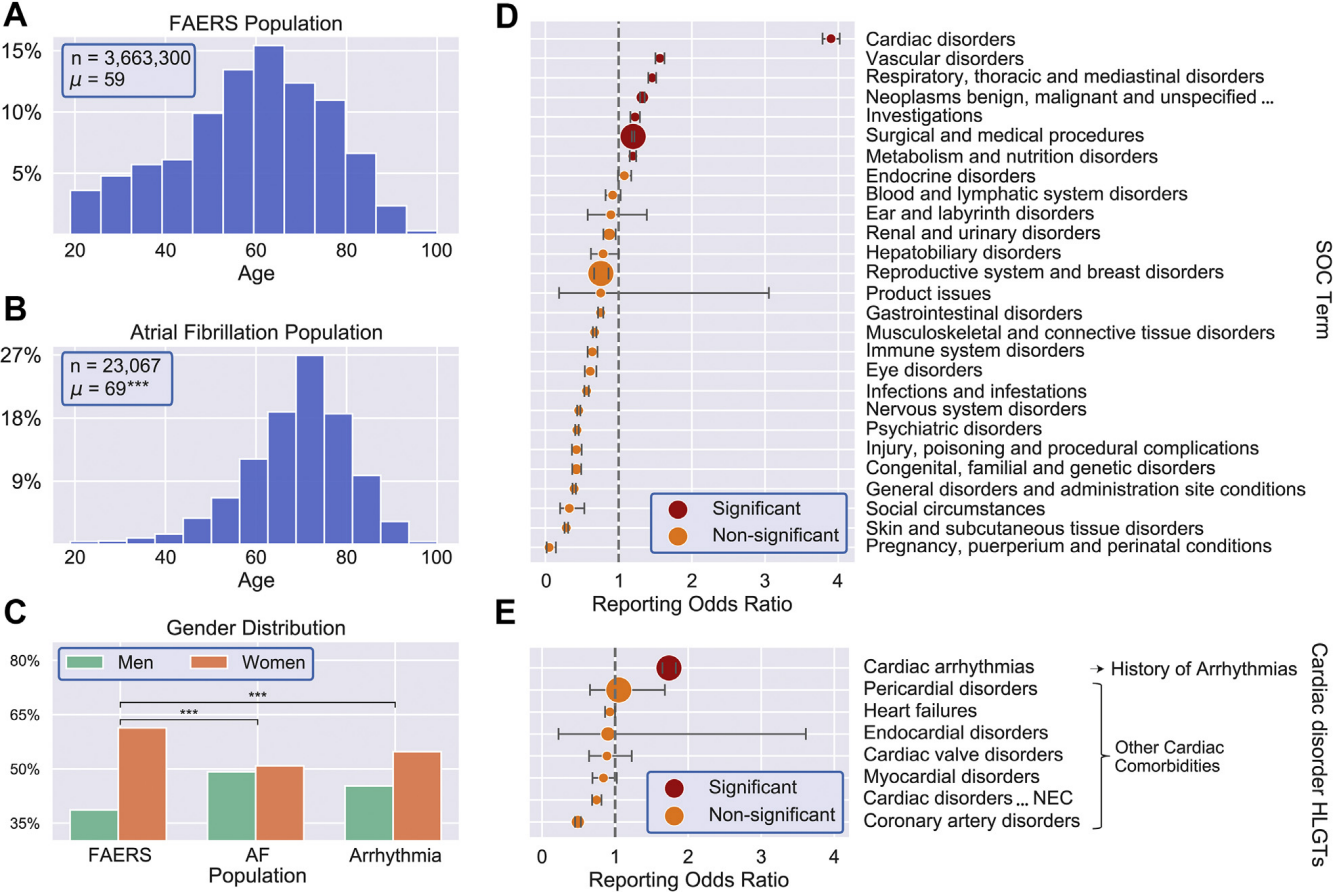
Identification of Confounding Effects **(A to C)** Summary statistics for age and sex of U.S. Food and Drug Administration Adverse Event Reporting System (FAERS) population and subpopulations. The age distribution for the FAERS population **(A)** and the subpopulation that reported atrial fibrillation (AF) **(B)** both tend to be elderly. *n* indicates number of cases in each population and *μ* is the population mean. The age distribution for the AF population is statistically significantly different from the background FAERS population (p < 0.001, Mann-Whitney *U* test; ∗∗∗p < 0.001, Student’s *t*-test). **(C)** Summary statistics for sex distributions for subpopulations affected by AF or any etiology of cardiac arrhythmia differ significantly from the background FAERS population (∗∗∗p < 0.001, Chi-squared test). **(D and E)** Analysis of comorbidities associated with AF reporting. **(D)** System organ class (SOC) level terms were analyzed by Chi-squared disproportionality testing to identify terms that co-segregated with elevated AF reporting. Significantly enriched terms, included as confounding variables in the final analysis, are shown in **red**. **(E)** AF reporting odds ratio (ROR) for the SOC level term “cardiac disorders” was large compared to the other terms and was analyzed for division at the high level group term (HLGT) stratum. “Cardiac arrhythmias” is significantly enriched (shown in **red**) over other cardiac disorders within AF reporting. RORs and confidence intervals for all terms are presented with Bonferroni corrections for multiple testing, and marker size is the proportional total number of reports containing the term.

The intersection between FAERS, MedDRA, and PKIDB allowed us to analyze the increased susceptibility to cardiac rhythm disorders subsequent to PKI treatment in a hypothesis-free fashion. There were significant population age differences when comparing populations with reported AF (median 70 years, 25th to 75th percentile: 62 to 77 years) or cardiac arrhythmia (median 63 years, 25th to 75th percentile: 45 to 73 years), compared with the general FAERS population (median 59 years, 25th to 75th percentile: 44 to 70 years; p < 0.001, Mann-Whitney *U* test) (Figures 1A to 1C). Similarly, the sex distributions were also significantly different between patients who developed AF or cardiac arrhythmia compared to the general FAERS population (p < 0.001, chi squared test). We also statistically evaluated which comorbidities needed to be considered as confounding effects (Supplemental Appendix). Several comorbidities aggregated by MedDRA hierarchy were enriched within AF reporting (Figure 1D). As cancer- and arrhythmia-related comorbidities are highly correlated with the treatment and outcome association that we are evaluating for, these confounding effects were accounted for separately (Figure 1E) (details in the Supplemental Appendix and Supplemental Figures 1 and 2). In the following analyses, we controlled for age, sex, and 6 independent aggregate comorbidities as confounding effects (history of arrhythmia, other cardiac (non-arrhythmia), respiratory, metabolism, vascular, and cancer).

### Seven PKIs increase reporting ORs of AF

**Figure 2:**
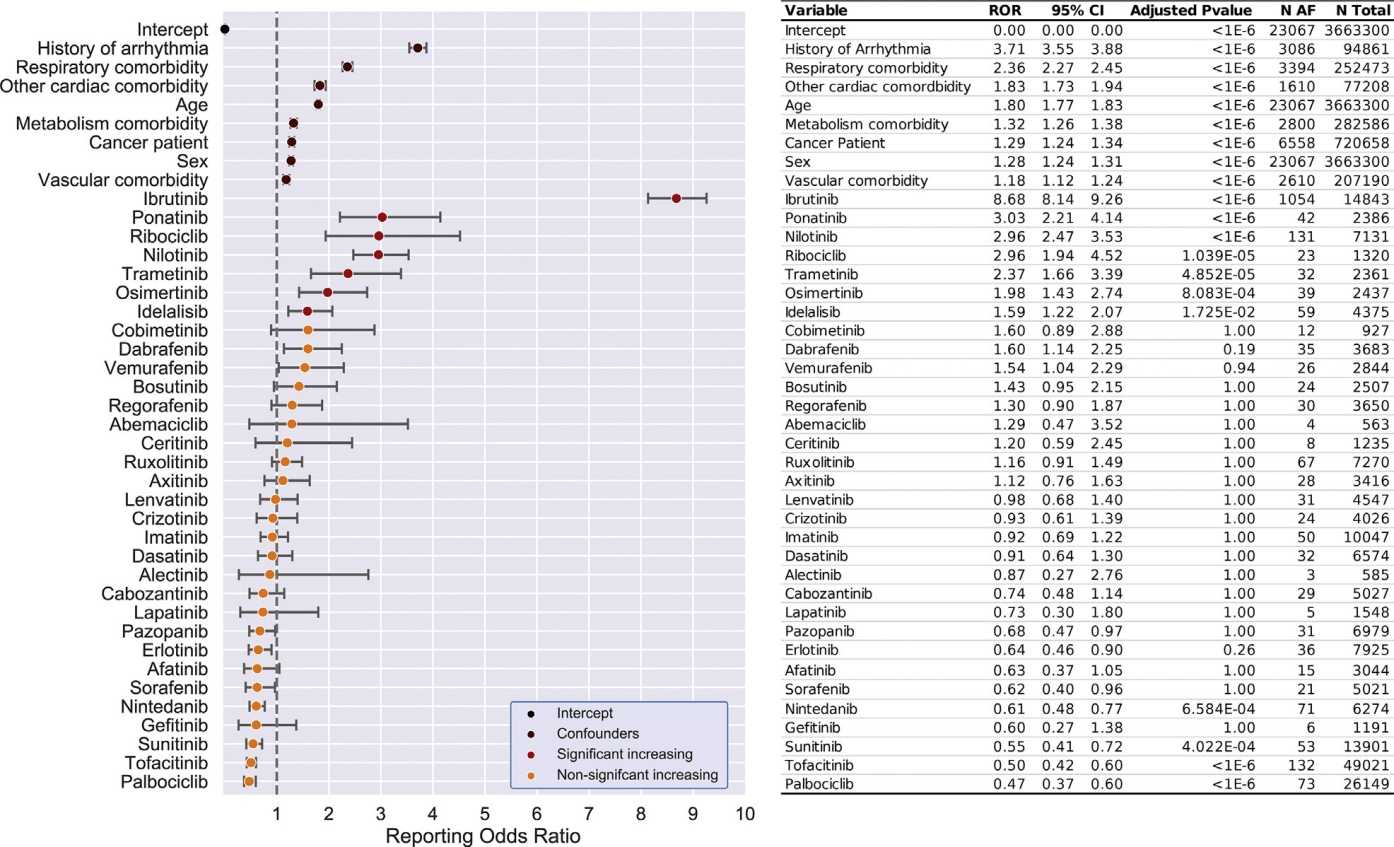
Odds Ratios for Protein Kinase Inhibitor Effect With Regards to Atrial Fibrillation Logistic regression analysis of AF in FAERS assessed the confounding effect size of comorbidities associated with increased incidence of disease, on the patient’s likelihood of developing AF (**dark red**). After controlling for these confounding variables, 7 protein kinase inhibitors have significantly increased likelihood of being reported for AF (**red**). These are: ibrutinib, ponatinib, nilotinib, ribociclib, trametinib, osimertinib, and idelalisib. The 95% confidence intervals (CIs) are skewed due to being in exponential space, and are not corrected for multiple hypothesis testing. Abbreviations as in Figure 1.

From the 3,663,300 adverse events reports that passed quality control, 23,067 patients were reported with AF. The effect of each of 32 PKIs on AF reporting was quantified using logistic regression. After adjusting for the confounding effects of age, sex, the 6 comorbidities identified, and multiple hypothesis testing rounds by Bonferroni-correction, our multivariable model identified 7 PKIs that significantly increased the risk of developing AF. These 7 oncotherapeutic PKIs included ibrutinib, ponatinib, nilotinib, ribociclib, trametinib, osimertinib, and idelalisib (Figure 2). Post hoc analysis did not identify any significant drug-drug interactions driving these findings (Supplemental Appendix and Supplemental Figure 3). Additional PKIs also had elevated ORs, such as vemurafenib and dabrafenib, but these did not achieve statistical significance after consideration for multiple testing. In this analysis, some PKIs were nominally associated with a reduced OR, but this is likely a spurious finding primarily linked to skewed AF reporting in the reference population (Supplemental Appendix and Supplemental Figures 4 and 5). Ibrutinib had an increased ROR of AF by almost 9-fold. The 6 other PKIs (ponatinib, nilotinib, ribociclib, trametinib, osimertinib, and idelalisib) increased the OR for AF by 2- to 3-fold.

### Proarrhythmic liability of PKIs beyond AF

**Figure 3:**
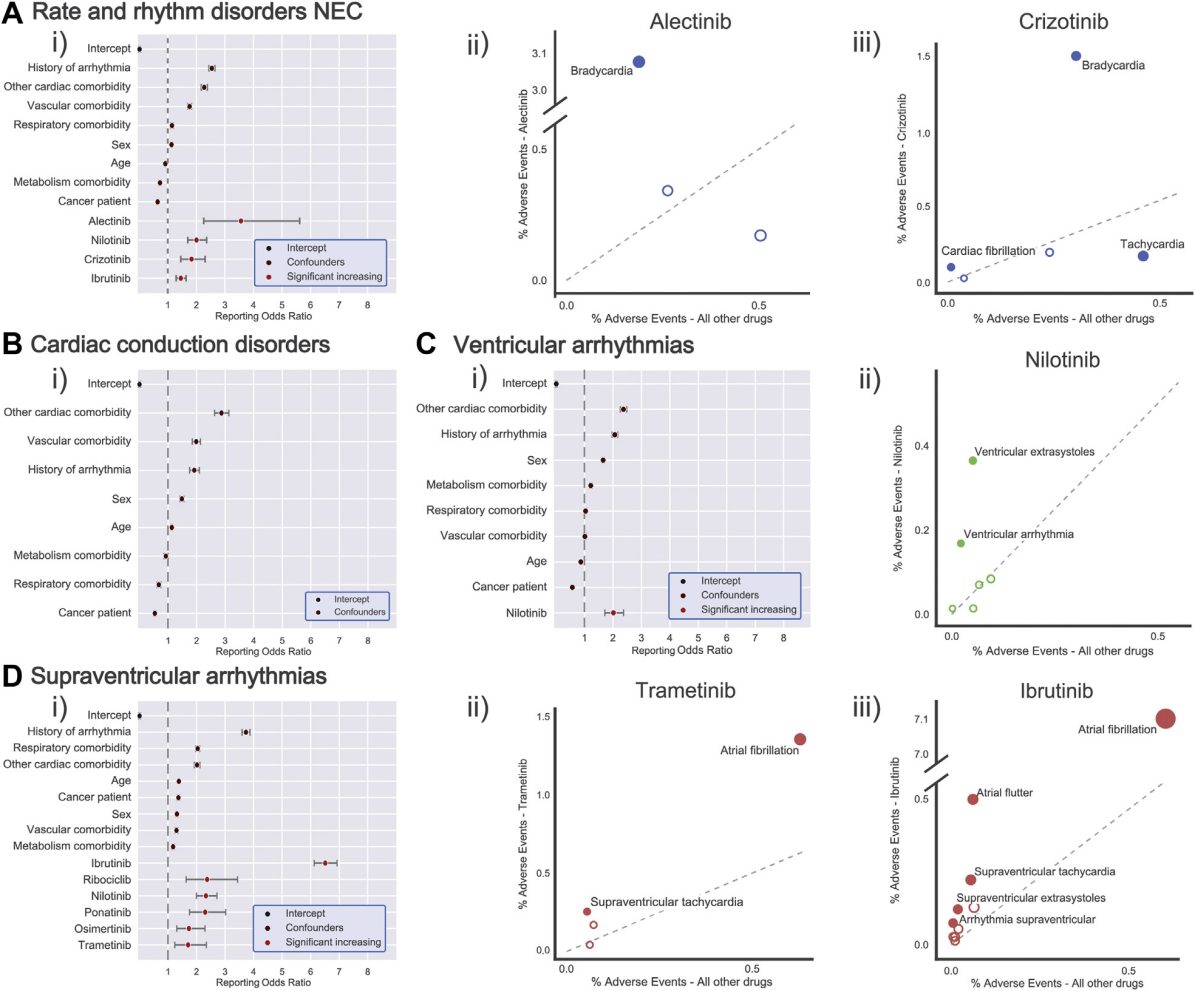
Odds Ratios for Protein Kinase Inhibitors With Regards to Related Arrhythmias All protein kinase inhibitors were assessed for elevated reporting of arrhythmias grouped under Medical Dictionary for Regulatory Activities (MedDRA) high level terms (HLTs): “rate and rhythm disorders” **(A)**, “cardiac conduction disorders” **(B)**, “ventricular arrhythmias” **(C)**, and “supraventricular arrhythmias” **(D)**. For each analysis, compounds with significantly elevated ROR after multiple-testing correction are presented along with confounder effects. Confounder effects with ROR < 1 does not imply a protective effect. For compounds with significantly elevated reporting, a post hoc analysis assessed for enrichment of underlying PTs to identify recurrent etiological themes. Significantly enriched or under-represented terms are labelled and shown with filled-in markers. The size of each marker is proportional to total number of reports for this form of arrhythmia for this drug. **(Ai)** Alectinib, nilotinib, crizotinib, and ibrutinib were associated with elevated rate and rhythm disorder reporting. The alectinib **(Aii)** and crizotinib **(Aiii)** signals were primarily driven by reporting of Bradycardia. **(Bi)** No protein kinase inhibitors were associated with elevated cardiac conduction disorder reporting. **(Ci)** Nilotinib was associated with elevated ventricular arrhythmia reporting. **(Cii)** This signal was primarily driven by reporting of ventricular extrasystoles and nonspecific ventricular arrhythmias. **(D)** With the exception of idelalisib, all other significant protein kinase inhibitors detected in the AF analysis were associated with elevated supraventricular arrhythmia reporting. Trametinib **(Dii)** and ibrutinib **(Diii)** signals were primarily driven by reporting of AF, and to a lesser extent by closely related terms (e.g., supraventricular tachycardia and atrial flutter). Abbreviations as in Figure 1.

We hypothesized that PKIs may predispose to arrhythmias beyond AF. We discovered that for the 7 PKIs significantly associated with AF, many other diverse arrhythmia types were also disproportionately reported (Supplemental Appendix and Supplemental Figure 6). To test this comprehensively, we analyzed whether there was an association between PKIs and cardiac arrhythmia reporting in general. As the MedDRA classification “cardiac arrhythmias” encompasses a broad range of diseases with variable etiologies, we subgrouped the 66,262 reports by the 4 MedDRA HLT classifications: “rate and rhythm disorders NEC,” “cardiac conduction disorders,” “ventricular arrhythmias,” and “supraventricular arrhythmias” for further analysis (the MedDRA abbreviation “NEC” denotes “Not Elsewhere Classified”). For each of these groups, all PKIs were assessed for elevated reporting, again controlling both for confounding factors and multiple testing. Several PKIs had elevated reporting for these disease groups (Figure 3). The specific arrhythmias under each HLT are conceptually related, but the clinical consequences can be diverse. Consequently, we performed post hoc analyses for each significant association to assess for etiological themes driving the association. Specifically, for each PKI with elevated HLT reporting, we assessed PT arrhythmia reporting relative to the general FAERS dataset by Chi-squared analysis. Select analyses are presented in Figure 3; the remaining analyses are in the Supplemental Appendix. Analysis of rate and rhythm disorders identified 4 compounds with significantly elevated reporting of arrhythmias: alectinib, nilotinib, crizotinib, and ibrutinib (Figure 3A). Alectinib and crizotinib signals were primarily driven by reporting of bradycardia (Figure 3Aii-iii). The enriched terms for nilotinib and ibrutinib do not suggest a specific etiology (e.g., “arrhythmia”) and may be primarily composed of atrial flutter and AF (Supplemental Figure 7A). Analysis of cardiac conduction disorders found no compounds with elevated reporting for these diseases (Figure 3B). For the confounding effects, ROR < 1 does not imply that a comorbidity is protective. Analysis of ventricular arrhythmias identified nilotinib as having significantly elevated reporting (Figure 3C). This association was driven by reports of ventricular extrasystoles as well as nonspecific ventricular arrhythmias (Figure 3Cii). Finally, analysis of supraventricular arrhythmias identified 6 of the PKIs as identified in the AF analysis above (Figure 3D). All supraventricular arrhythmia associations were primarily driven by AF (Supplemental Figure 7B), and trametinib and ibrutinib were notably also supported by closely related terms (e.g., supraventricular tachycardia and atrial flutter [Figure 3Dii-iii]). Compared to the AF analysis, the ORs in this analysis were lower indicating that grouping of other supraventricular arrhythmia phenotypes actually dilutes the exceptionally large association of these kinase inhibitors with AF. The analyses presented here identified increased ORs for reported bradycardia for alectinib and crizotinib, and increased ORs for reported ventricular arrhythmias for nilotinib. These findings are summarized in the Central Illustration.

**Central Illustration:**
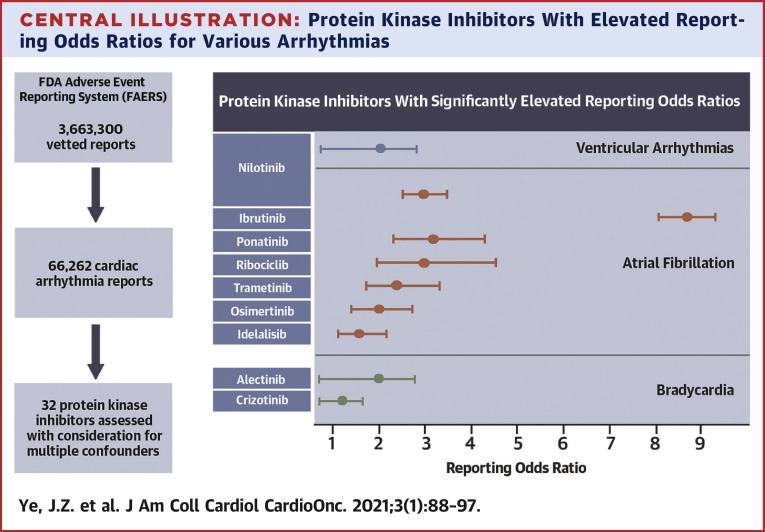
Protein Kinase Inhibitors With Elevated Reporting Odds Ratios for Various Arrhythmias Logistic regression analysis of 3,663,300 U.S. Food and Drug Administration (FDA) Adverse Event Reporting System (FAERS) reports (66,262 cardiac arrhythmia reports) that assessed the likelihood of developing arrhythmia. After controlling for confounding variables, 9 protein kinase inhibitors have significantly increased likelihood of being reported for arrhythmia, including ibrutinib, ponatinib, nilotinib, ribociclib, trametinib, osimertinib, and idelalisib for atrial fibrillation specifically. The 95% confidence intervals are skewed due to being in exponential space, and are not corrected for multiple hypothesis testing.

## Discussion

In recent years we have witnessed an increasing number of reports on cardiotoxic effects of the tyrosine kinase inhibitor ibrutinib, as well as other tyrosine kinase inhibitors such as ponatinib and osimertinib (2, 3, 4, 5,17,18). Here we set out to evaluate if arrhythmogenic side effects are a common phenomenon for PKIs used in cancer therapy, which would necessitate additional attention to this potential toxicity within this class of pharmacologic agents. To this end we examined the FAERS and comprehensively evaluated the reporting rates of cardiac arrhythmias from use of kinase inhibition treatments. We identified 7 PKIs that increase reporting odds for AF, 2 that increase odds for bradycardia, and 1 associated with increased risk for ventricular arrhythmia. Three of these PKIs have been previously reported as having an association with increased AF risk, whereas another recent report also finds an additional AF association with nilotinib (2,4,5,19). We present novel associations with AF for ribociclib (previously associated with prolonged QT), trametinib, and idelalisib (20). Additionally, we present associations of alectinib and crizotinib with bradycardia and additional ventricular proarrhythmic liability for nilotinib. The primary targets of these inhibitors are different protein kinases, but they all have additional off-target effects. It remains to be discovered which collection of kinases and pathways are critical to elicit these proarrhythmic responses. An important next step is to identify on- and off-target kinase signaling pathways, especially those modified by multiple agents, to determine which are driving the arrhythmogenic effects. With this knowledge, further drug development can aim to minimize activation of these pathways.

### Effects of age is lower than anticipated due to skewed age distribution in FAERS

In our multivariable model, age contributed less to the probability of developing any AF (ROR: 1.80; 95% CI: 1.77 to 1.84) than initially expected (21). We attribute this to the skewed population in FAERS, which is more elderly than the general population, and intrinsically samples from a less-healthy portion of the population within each age group, resulting in a potentially biased sample (22). Consequently, the apparent prevalence of disease in the younger proportion of the FAERS population may be higher than the general population, and possibly attenuate the relationship between AF and age (21). The relationship with age was weaker still in the analysis of the HLT-grouped arrhythmias. We hypothesize that this is related to other disease etiologies being more localized and developing more acutely (e.g., sinus bradycardia or extrasystoles), and thus less age-dependent than AF which requires broader tissue remodeling to create a proarrhythmic substrate capable of supporting a re-entrant arrhythmia.

### Effects of comorbidities

Comorbidity effect sizes in the logistic regression models were largely similar to those in the Chi-squared analysis. Minor differences are likely attributable to reports with multiple comorbidities which are assessed independently in the Chi-squared analysis but simultaneously by logistic regression. In the results presented in Figure 3 it can be observed that some of the comorbidities present with an ROR <1. In this analysis the comorbidity confounder effects quantify the frequency with which arrhythmic adverse events occur relative to all other adverse events in the context of a specific comorbidity, and furthermore, how this relative frequency for the comorbidity group compares to the relative frequency within a reference population. Here, the reference population is a patient population being treated for diverse diseases with various drugs; many of which have well-recognized proarrhythmic liabilities (e.g., dofetilide and digoxin). Consequently, the global population of the database is enriched for arrhythmic adverse events. As a result, it is possible that within a subpopulation, those reporting a “metabolism and nutrition disorder,” for example, are enriched for nonarrhythmic events relative to the arrhythmia-enriched global FAERS population. As such, confounder variables with an ROR <1 do not imply that a comorbidity is protective against arrhythmias. Rather, it implies that the drugs used to treat patients with the specific comorbidity more frequently result in adverse events other than the collection of arrhythmias being studied when compared to this relative frequency for all other drugs in the FAERS database combined.

### Protective effects overestimated due to skewed reference population

An oft-ignored aspect during signal detection in pharmacovigilance studies is that multiple drugs are spuriously found to significantly decrease ORs, as also observed in our study. In our case we cannot conclude that certain PKIs in our study, such as tofacitinib, palbociclib, and sunitinib, are truly protective against developing arrhythmias. The logistic regression calculates the odds compared to a reference population. The reference population in our model also comes from the FAERS database, and as such all are receiving medical attention and have experienced one or more adverse event. As the baseline for comparison is itself not a healthy population, the presence of comorbidities is likely to increase the basal incidence of disease relative to the population at large. Furthermore, the reference population likely has an inflated arrhythmia rate attributable to reports for drugs with known proarrhythmic liability. The Supplemental Appendix results present an analysis that partially corrects for this, showing that the nominally protective PKIs in the conservative analysis above are likely spurious. Although we show that these protective effects may be exaggerated in the chosen multivariable model, a similar exercise could be done using a disproportionality analysis. Together, this highlights that apparent protective effects of PKIs or comorbidities cannot be concluded from this analysis.

### Seven tyrosine kinase inhibitors significantly predispose for heart rhythm disorders

Conversely, we identified 9 PKIs that significantly increase the OR of various cardiac arrhythmias (ibrutinib, ponatinib, nilotinib, ribociclib, trametinib, osimertinib, idelalisib, alectinib, and crizotinib) (Central Illustration). This finding was made even when comparing to an elderly, unwell population, suggesting that the true ORs might be greater if compared to a more representative control group. Seven of these PKIs target tyrosine kinases (ibrutinib, ponatinib, nilotinib, trametinib, osimertinib, alectinib, and crizotinib). This is particularly interesting, as our knowledge of the functional role of tyrosine kinases in the adult heart is limited and suggests that tyrosine protein kinases may play a hitherto unrecognized role in cardiac rhythm maintenance. Five of the tyrosine kinase inhibitors exert a much greater effect that results in AF compared to other cardiac arrhythmias. This may indicate that the pathways regulated by these tyrosine kinase inhibitors specifically predispose to AF, but there are general enrichments across all types of arrhythmias (Supplemental Appendix). This is certainly the case for ibrutinib (4,19). Our results fall closely in line with other studies of PKIs and other large-scale pharmacovigilance studies using disproportionality analysis methods (2,4,5,17,18). The molecular mechanisms by which inhibition of a tyrosine kinase leads to arrhythmia are yet to be identified. Investigations into yet uncovered shared targets and shared biological pathways will be needed to elucidate the molecular underpinnings of the arrhythmogenic mechanism of tyrosine kinase regulation.

### Study limitations

This study retrospectively analyzed the adverse events included in FAERS, using data from the database as a baseline. Consequently, the analysis outcome is highly dependent on the data quality included in FAERS. We identify 2 main sources of bias in our study. First, the ORs for each drug are reflective of the pharmacological effects on physiology, but may be subject to underreporting as attribution and reporting of adverse events is filtered by how closely patients are followed and awareness within the patient population of how potential side effects could present (23,24). The most commonly prescribed kinase inhibitors are likely to have more total adverse event reports of greater variety and diminished clinical veracity due to reports submitted by non–health care providers. Secondly, our analysis may be affected by potential bias with missing data. Missing values can be divided into 3 categories; namely, missing completely at random, missing at random, and missing not at random. We have treated the missing values in age and sex as missing completely at random and excluded reports with missing values from the final analysis, allowing us to train our model without considering any potential latent structure to the missingness. However, the true relationship may be different. Another potential limitation is that there may exist duplicate reports beyond the 46,394 reports we identified by matching report information. Finally, all results reported are dependent on the reporter being able to correctly identify the primary source of the adverse event, and only reporting relevant phenotypes as adverse reactions. Because of these limitations, we emphasize that our results indicate an association between the PKIs and cardiac arrhythmia; however, inferring causal relationship and quantifying the true relative risk of these agents requires further prospective studies and functional research.

## Conclusions

Seven PKIs (ibrutinib, ponatinib, nilotinib, ribociclib, trametinib, osimertinib, and idelalisib) are associated with an increased relative risk of developing AF. Nilotinib has an additional ventricular-specific proarrhythmic liability, whereas bradyarrhythmic-specific liability exists for alectinib and crizotinib. Additional monitoring for cardiac complications in patients receiving these compounds should be considered. Common protein kinase signaling pathways by these compounds should be prioritized for investigation into the mechanisms behind PKI-associated cardiotoxicity.

Perspectives **COMPETENCY IN MEDICAL KNOWLEDGE:** The PKIs ibrutinib, ponatinib, nilotinib, ribociclib, trametinib, osimertinib, and idelalisib were each associated with an increased ROR for AF. Alectinib and crizotinib were associated with an increased ROR for bradycardia and nilotinib with additional ventricular proarrhythmic liability.

**TRANSLATIONAL OUTLOOK:** Additional research is necessary to determine the mechanisms underlying cardiotoxicity associated with these PKIs. An important next step is to identify on- and off-target kinase signaling pathways, especially those modified by multiple agents, to determine which are driving the arrhythmogenic effects. With this knowledge, further drug development can aim to minimize activation of these pathways.

## Funding Support and Author Disclosures

Supported by the Novo Nordisk Foundation grant NNF20OC0059767 to Dr. Lundby. The authors have reported that they have no relationships relevant to the contents of this paper to disclose.
