# Supplementary material for: Oncotherapeutic Protein Kinase Inhibitors Associated With Pro-Arrhythmic Liability
Source: JACC CardioOncol. 2021 Mar 16;3(1):88–97. doi: 10.1016/j.jaccao.2021.01.009 (PMC8352262; doi:10.1016/j.jaccao.2021.01.009)
Supplement: Supplemental Data [file mmc1.docx]

**SUPPLEMENTAL MATERIAL**

### DATA SOURCING AND VETTING

### Protein Kinase Inhibitor Database (PKIDB)

Protein kinases are a class of proteins that consists of more than 500 different enzymes that catalyze phosphorylation of serine, threonine or tyrosine residues on target proteins, which frequently leads to protein activity regulation. Many kinases play essential roles in signal transduction and cell cycle control [1] making them targets of oncotherapeutics. The Protein Kinase Inhibitor database [2](PKIDB) is updated monthly and collates information on 212 (visited 1 September 2019) different protein kinase inhibitors used clinically or in clinical trials for cancer and other disease treatments.

### FDA Adverse Event Reporting System (FAERS)

To analyze the unintended proarrhythmic effects of protein kinase inhibition, we collated and assessed reports from publicly available adverse event cases from the FDA adverse events reporting system (FAERS – visited 21. August 2019) [3]. This database is updated quarterly and contains adverse events reports primarily from the United States of America. Reporting is mandatory for drug manufactures, but may also be submitted by healthcare professionals as well as consumers (patients, relatives, lawyers etc.). In addition to patient and other administrative data, such as submitter classification, each report also includes information about drugs administered, adverse events observed, treatment and outcome, and suspected causative drug when multiple agents are used concurrently. We examined the data in FAERS from the five-year period: 2014Q3-2019Q2, during which 6,953,042 individuals had reported cases of adverse events and medication errors. Previous to this period reports were not annotated with generic drug names, and as such were excluded to maintain high data quality. The reports were rigorously vetted; only reports where the suspected causative agent of the adverse event is indicated were included, retaining only entries with values for age in the range 18-100 years and gender specified as male or female, such that every included report was for an adult patient and had complete data to allow for analysis of confounding effects. Similarly, only drugs with more than 500 vetted adverse event reports in FAERS were included to ensure appropriate statistical power for evaluation, resulting in 32 protein kinase inhibitors assessed. For the assessed inhibitors, all reports dated prior to their FDA approval were excluded to ensure consistent drug exposures. The latter resulted in exclusion of 608 reports, the vast majority (556) for Cabozantinib. We excluded 46,394 reports identified as duplicate reports by matching information pertaining to event date, manufacturer date, report type, authority number, manufacturer number, literature reference, age, age unit, age group, gender, electronic submission, weight, weight unit, voluntary report, report occupation, reporter country, and occurrence country. After this thorough vetting, the final analysis contained 3,663,300 reports.

### Medical Dictionary for Regulatory Activities (MedDRA)

The Medical Dictionary for Regulatory Activities (MedDRA) is an international medical dictionary categorizing diseases into five different hierarchical disease groups: a) System Organ Classes (SOC); b) High Level Group terms (HLGT); c) High Level Terms (HLT); d) Preferred Term (PT); and e) Lowest Level Term (LLT). The FDA utilizes MedDRA to standardize and classify reported adverse events [3]. ‘Cardiac arrhythmias’ is considered a HLGT by MedDRA and contains 112 LLTs. 'Atrial fibrillation’ is an LLT/PT in MedDRA and part of the HLGT ‘cardiac arrhythmias’. We excluded five LLTs: ‘Cardio-respiratory arrest’, ‘Cardiac arrest’, ‘Sudden death’, ‘Sudden cardiac death’, and ‘Pulseless electrical activity’, as these terms are heavily associated with ontologies outside cardiac rhythm disorders, leaving a total of 107 LLTs.

### CONFOUNDING EFFECTS

### Age and Gender are Confounding Effects

It is well-established that age and gender affect the risk for developing cardiac arrhythmias. Accordingly, we evaluated the need to correct for potential age and gender discrepancies in the study population by comparing distributions of patients with cardiac rhythm adverse events against age and gender distribution of the remaining patients in the FAERS database. As expected, we found significant population age differences and gender distributions differences between patients who developed atrial fibrillation or cardiac arrhythmia compared to the general FAERS population, and accordingly it is necessary to compensate for confounding effects by age and gender. Thus, the probabilistic effect of all protein kinase inhibitors should be evaluated using a multivariable model. We chose a logistic regression, with age as a continuous variable and gender as a bivariate categorical variable.

### Comorbidities Identified as Confounding Effects

We evaluated for additional confounding effects contributing to atrial fibrillation reporting by assessing co-segregation of comorbidity terms. MedDRA standard descriptor terms present in the ‘Indications’ section of reports were clustered by SOC hierarchy and analyzed by χ2 testing for association with atrial fibrillation reporting across the general FAERS population. Comorbidities clustered under ‘Cardiac disorders’, ‘Vascular disorders’, ‘Respiratory, thoracic and mediastinal disorders’, ’Neoplasms benign, malignant and unspecified’ and ‘Metabolism and nutrition disorders’ each had significantly elevated atrial fibrillation reporting when independently compared to all other terms combined (as shown in Figure 1D). The identification of the SOC term ’Neoplasms benign, malignant and unspecified’ could reflect the observation that cancer patients appear more prone to developing atrial fibrillation than the general population independent of treatment-associated cardiotoxicity [4]. However, as we are evaluating oncotherapeutic compounds for potential treatment-associated pro-arrhythmic liability in excess of disease-associated liability, these two effects require parsing as compared to the other confounding effects where disease- and treatment-associated pro-arrhythmic liabilities are aggregated. From a curated list of 178 oncotherapeutics, χ2 analysis conservatively identified six protein kinase inhibitor and 14 non-protein kinase inhibitor compounds with disproportionate atrial fibrillation reporting within the cancer-treated patient population. This was adjusted for in the implementation of the treatment-independent ‘Cancer Patient’ confounding factor (details in section below). In the results shown in Figure 1D, the SOC term ‘Cardiac disorders’ had a much greater odds ratio for reported atrial fibrillation than the other terms and was thus assessed for division at the High Level Group Term stratum. That revealed that the signal was disproportionately driven by the HLGT, ‘Cardiac Arrhythmias’ (as shown in Figure 1E), and as such ‘Cardiac disorders’ was partitioned into two independent factors: 'History of Arrhythmia' and ‘Other Cardiac Comorbidity’. Consequently, the analysis of association of protein kinase inhibitors with atrial fibrillation was controlled for the presence of the six comorbidity confounding factors identified: ‘History of Arrhythmia’, ‘Other Cardiac Comorbidity’, ‘Vascular Comorbidity, ‘Respiratory Comorbidity’, ‘Metabolism Comorbidity’, and ‘Cancer Patient’ (details below).

### Treatment-Independent ‘Cancer Patient’ Confounding Factor

In the section above, we describe the identification of comorbidities clustered under ’Neoplasms benign, malignant and unspecified’ as a confounding effect contributing to atrial fibrillation adverse event reporting. As we are evaluating oncotherapeutic compounds for potential treatment-associated pro-arrhythmic liability in excess of disease-associated liability, these two effects require parsing. There is some evidence that cancer patients are more prone to developing atrial fibrillation (AF) than the general population independent of treatment-associated cardiotoxicity [5]. As such, we included a separate confounding factor to account for any specific increased AF liability as a consequence of being a cancer patient but independent of cancer-treatment associated liability. First, we identified the sub-population of reports in FAERS belonging to cancer-treated patients. A report was classified as belonging to a cancer patient if the report listed any drug from a curated list of cancer drugs [6]. All reports where the patient was indicated as receiving any of the 178 identified compounds were isolated from the FAERS database; restricting the approach to the same time period and filtering resulting in 111 compounds with at least 500 vetted reports. This sub-population of FAERS reports is internally skewed with an elevated proportion of reports for atrial fibrillation and other cardiac arrhythmias due to compounds with established cardiotoxicity resulting in pro-arrhythmic liability, e.g. melphalan and lenalidomide [7]. In order to accurately capture the pro-arrhythmic liability associated with disease alone and not that associated with treatment, compounds that disproportionally skew AF reporting within the cancer patient population need to be identified and excluded from the confounding variable. To determine which compounds skew this population, reporting for each anti-cancer compound was individually compared to the combined reports for all other anti-cancer drugs.

First, we noted that Ibrutinib is an extreme outlier with disproportionate influence on the population. Specifically, when comparing the proportion of reported adverse events that concern atrial fibrillation (AF proportion) for each compound, Ibrutinib is a significant outlier from the group (Supplemental Figure 1A). A jackknife procedure was used to estimate the variability of the weighted-mean of AF proportion, wherein each compound’s contribution to the mean is weighted by the total number of reports for that compound. In the jackknife procedure for the weighted mean, the weighted mean is calculated repeatedly, each time leaving out one of the compounds producing a distribution of estimates of the weighted mean. The weighted mean wherein Ibrutinib is excluded is a significant outlier from the distribution (Supplemental Figure 1B). Furthermore, re-calculating the jackknife means but systematically excluding Ibrutinib from the group results in a significantly shifted distribution. In contrast, additional systematic exclusion of the other outlier compounds does not significantly shift the jackknife means distribution. Together this demonstrates that the reports for Ibrutinib uniquely exert exceptional skewing influence on the descriptive statistics of the anti-cancer compounds, and as such Ibrutinib reports were excluded prior to comparing the individual anti-cancer compounds against the group.

After *a priori* exclusion of Ibrutinib, we compared each anti-cancer drug against the combined group of anti-cancer drugs by χ^2^ disproportionality analysis. Individually comparing AF reporting of each anti-cancer compound against the group identified an additional 5 PKI and 14 non-PKI anti-cancer compounds out of 110 compounds as having significantly elevated reporting odds ratios (Supplemental Figure 2). These compounds were thus excluded from the confounding variable for the subsequent analysis of AF reporting for PKIs. It should be noted that this vetting is still conservative as additional compounds with recognized AF liability, e.g. cyclophosphamide [7], remain within the confounding variable even with elevated reporting odds ratio. After removing the highly disproportionate compounds, the AF reporting proportion for onco-therapeutic compounds was 0.84% as compared to 0.59% in the general FAERS database. The analysis above identified 6 PKI onco-therapeutics (including Ibrutinib) with significantly elevated AF reporting relative to the aggregate group, and as such these PKIs are excluded from the evaluation of the treatment-independent Cancer Patient confounder effect size. However, reporting for these 6 PKIs must still be controlled for this confounding effect. These two goals cannot be accomplished with a simultaneous regression of AF reporting for the Cancer Patient confounder and the 6 PKIs. Consequently, the effect sizes for all confounding factors were simultaneously assessed through logistic regression of AF reporting for all drugs within the FAERS database but with reporting for the 20 compounds that skew the treatment-independent Cancer Patient confounder variable removed. Subsequently, these reports were returned to the dataset and every report throughout the dataset was individually controlled for its internal composition of confounders during a secondary logistic regression that assessed the confounder-controlled independent effect size of each of the PKIs on AF reporting [8]. Every report concerning a protein kinase inhibitor was controlled for the ‘Cancer Patient’ confounder with the exception of reports for Tofacitinib as this PKI is not an onco-therapeutic.

### POTENTIAL DRUG-DRUG INTERACTIONS IN ATRIAL FIBRILLATION REPORTING

In the primary text, we describe seven protein kinase inhibitors (PKIs) with significantly increased reporting odds ratios (ROR) for atrial fibrillation (AF). It is the purpose of this work to detect the broader pro-arrhythmic liability of these compounds, which will allow us to cross-reference the many pathways variably affected by each drug with the goal to unravel which proarrhythmic pathways are being activated. With this knowledge, further drug development can aim to minimize activation of these pathways. In general, drug-drug interactions would act to “sensitize” patients to potential pro-arrhythmic effects, e.g. CYP3A modifiers resulting in elevated serum levels of the compound resulting in increased incidence of atrial fibrillation is part of the liability we attempt to detect. However, as it is important for clinical considerations to know if these liabilities result in arrhythmia *primarily* in the context of a sensitizing agent, we performed post-hoc analyses of co-administered compounds. For each of the seven PKIs with significantly elevated AF reporting, report-IDs were identified where the PKI was listed as Primary Source of the adverse reaction, and these reports were divided by whether or not ‘Atrial fibrillation’ was listed as an adverse reaction. The Drug File was then filtered using these two ID lists to identify and count (once per primary ID) any drugs co-administered with the PKI. χ^2^ disproportionality testing was used to assess the AF reporting for each PKI/co-administered drug combination relative to reporting for the PKI administered alone. Only Ibrutinib and Nilotinib had significantly elevated reporting for co-administered drugs (Supplemental Figure 3). These co-administered drugs associated with elevated reporting relative to the PKI administered alone were anti-arrhythmic, anti-coagulant, anti-hypertensive, and diuretic medications. As these drugs typically are treatments for conditions, symptoms, and sequelae of conditions already associated with increased propensity for new episodes of AF, these associations are not suggestive of drug-drug interactions driving new-onset AF. Rather, these are effects already controlled for in our analysis by the ‘History of Arrhythmia’ and ‘Other cardiac comorbidity’ confounder variables.

### APPARENT PROTECTIVE EFFECT OF SOME PROTEIN KINASE INHIBITORS IS DRIVEN BY SKEWED REFERENCE POPULATION

In the results presented in Figure 2, some protein kinase inhibitors had reporting odds ratios less than one, and thus appear to present protective effects against atrial fibrillation. We inferred that these signals are likely artifacts of the reference population used. In the analysis we performed, the odds for reported new-onset arrhythmia attributed to protein kinase inhibitor use were compared to the odds within the reference population comprised of the pooled reports from all non-protein kinase inhibitor drugs within the FAERS database. However, this population includes reports of drugs with known pro-arrhythmic liability, such as dofetilide and digoxin, and may include others where this liability exists but is not yet recognized. This would be expected to inflate the reference arrhythmia rate. In an attempt to demonstrate a more comprehensive analysis, we applied bootstrap population resampling techniques to reduce the influence of pro-arrhythmic compounds in the reference population and repeated the atrial fibrillation analysis presented in Figure 2 of the primary text. (detailed methods below, Supplemental Figure 4). Performing the analysis with adjusted reference populations caused the odds ratios for all protein kinase inhibitors to be higher (Supplemental Figure 5). Compound confidence intervals show how the uncertainty with which the background disease report rates are estimated can influence the resulting odds ratios. When combined with the standard confidence intervals for the regression these effects essentially eliminate the apparent protective effect displayed by some of the protein kinase inhibitors in the primary analysis. That is, the analysis suggests that the protein kinase inhibitors nominally associated with a reduced odds ratio in Figure 2 is a spurious finding primarily linked to a skewed background population.

### Presence of Pro-arrhythmic Compounds in the Reference Population

The association of protein kinase inhibitors (PKIs) with atrial fibrillation or other cardiac arrythmias is characterized as the odds ratio of reported new-onset disease attributed to PKI use compared to reported new-onset disease within the reference population. The reference population consists of the pooled reports for all other non-PKI drugs reported to the FAERS database and vetted as described. This pooled-report reference disease rate includes compounds with known pro-arrhythmic liability, e.g. digoxin, which would be expected to inflate the arrhythmia reporting rates in the reference population. Lacking an objective means to determine which drug-reports should be excluded from the reference population, we chose the most conservative approach in the analysis presented in the main text. In this conservative approach, atrial fibrillation represented 0.59% of adverse reports represented in the background population. In the logistic regression analysis of AF reporting, the adverse events for the individual PKIs are in-effect compared against these rates, after accounting for confounding variables. When disease reporting rate in the reference population is inflated, then PKIs that have no effect on disease incidence will have a reported rate lower than this inflated reference rate, and thus can appear to be ‘protective’ against disease in the conservative analysis. To evaluate if this is the case, we performed an analysis using population resampling techniques to objectively construct a reference population with reduced influence of pro-arrhythmic compounds. The details are described below.

### Estimation of Reference Disease Reporting Rates by Weighted Medians

When disease reporting rates are calculated for each compound individually (atrial fibrillation reports as a percentage of all adverse event reports for the specific drug) and their distribution plotted, this distribution is heavily skewed (Supplemental Figure 4A). Robust measures of distribution central tendency are more appropriate for skewed distributions, for example using the median over the mean. However, rather than evaluating standard medians, the number of cases for each drug should also be considered. For example: the cardiac arrhythmia reporting rate for digoxin (43%) should not contribute equally to these calculations as the rate for etanercept (0.4%), as there are 1085 reports for digoxin and more than 200,000 reports for etanercept. To compensate for this, we weight the drug-specific reporting rates by the total number of reports for the drug. If we were to calculate the weighted mean for the distribution, we would recover the pooled-reports rate present in the conservative approach analysis of the main text specified above. However, when calculating the more robust weighted median, we find that atrial fibrillation represents 0.36% of adverse reports in the background population. This metric provides an estimate of the background disease reporting rate with reduced influence of pro-arrhythmic compounds and could be used to perform an adjusted logistic regression of the reporting data as described below. However, the weighted median is a summary statistic of a distribution, and all such statistics have a degree of uncertainty to the value, which is typically expressed as a confidence interval for the parameter. As the specific value of the reference disease rate in the logistic regression analysis can have significant influence on the exact value of the odds ratios estimated by the model, and thus significantly influence the conclusions reached for any particular PKI, this uncertainty of the weighted median value must be incorporated into the analysis. To evaluate the uncertainty for each of these metrics we turn to bootstrap estimation.

### Bootstrap Estimation of Confidence Interval for Reference Disease Reporting Rates

### To estimate the uncertainty surrounding the weighted-median measure of disease reporting rates we apply the bootstrap resampling method. The principle is that we generate numerous same-size derivative populations by sampling with replacement from the elements of the original dataset. From each of these derivative populations we re-estimate the weighted median, which will be slightly different for each derivative population. Gathering these many variant re-estimates of the weighted median, we can calculate the mean and percentile confidence interval of the weighted median re-estimates. In this instance, derivative populations were constructed by sampling with replacement, but the likelihood of a particular drug’s disease reporting rate being sampled was weighted in proportion to the number of reports for the drug, after which a standard median was calculated. This results in less-biased derivative populations than using un-weighted resampling of the reporting rates and then calculating a weighted-median. 100,000 such re-estimates of the weighted-median reporting rates were made, giving rise to distributions of the statistic which is nearly normally distributed after bootstrap smoothing (Supplemental Figure 4B). Smoothing is used as the median of each derivative population can be only a limited number of discrete values (an actual value from within each derivative population as the population size is odd) resulting in a discontinuous distribution. This was overcome through the process of bootstrap smoothing [9], wherein a small amount of random Gaussian noise perturbations are added to the individual elements of the derivative populations prior to calculating the median values. The Gaussian noise had a mean of zero and standard deviation equivalent to the standard error of the mean, which was in turn estimated as the standard deviation of the means from a secondary set of 100,000 bootstrap populations created as above. From the final smoothed bootstrap distribution, wherein each element is a number-of-reports weighted median of reporting rate, the mean and 99.85% confidence interval tail values were calculated (Supplemental Figure 4B). This confidence interval (CI) is a 95% confidence interval after adjusting for multiple comparisons using the Bonferroni correction for the 32 PKIs being tested. This analysis provides an estimate of the background disease reporting rates with reduced influence of pro-arrhythmic compounds, but further, it also provides an uncertainty interval around these estimates. These values were used to perform adjusted logistic regression of the reporting data as described below.

### Adjusted Reference Population Re-Analysis of PKI Associations with AF

The adverse events reporting data was re-analyzed by logistic regression as described in the main text, but the reference population was adjusted to reflect the median background reporting rates for atrial fibrillation and associated uncertainty intervals estimated above. To objectively adjust the reference population, the non-PKI reports were randomly sampled to construct a new reference population, but the random sampling was constrained to reproduce a specific pooled disease reporting rate. For example, all non-PKI drug reports were gathered into two pools, one for those regarding atrial fibrillation and one for all other adverse events. These two pools were randomly sampled with replacement to construct an adjusted reference population of the same original size but 0.36% of reports came from the atrial fibrillation pool, reflecting the mean weighted-median value found above. Although not expressly controlled for, it is expected that the random sampling process from large pools generates an adjusted population with age, gender, and other confounding variable composition that closely approximates that of the original due to the large number of samples. The logistic regression was performed with this adjusted reference population, and the resulting odds ratios reported as the central filled circles in Supplemental Figure 5. Two additional adjusted reference populations were similarly constructed but with pooled reporting rates constrained to one of the bootstrap confidence interval tail-value reporting rates from above. Regressions using these reference populations result in an uncertainty interval bracketing the central odds ratio, presented as filled triangles in Supplemental Figure 5. Compounded on this interval is the standard uncertainty from regressing a model onto real data to derive model parameters, presented as the outermost bars in Supplemental Figure 5. This outermost interval is the compound multiple-testing adjusted confidence interval (99.85%) for the odds ratio.

The reduced background disease reporting rates in the adjusted reference populations caused the odds ratios for all PKI’s to be higher for atrial fibrillation (Supplemental Figure 5). Compound confidence intervals show how the uncertainty with which the background disease reporting rates are estimated can influence the resulting odds ratios. These effects when combined with the standard confidence intervals for the regression result in eliminating the apparent protective effect displayed by some of the protein kinase inhibitors in the conservative analysis. This approach demonstrates that the apparent protective effects of some PKIs, as represented in Figure 2 of the primary text, cannot be concluded from this study as these effects are most likely an artifact of the reference population. Conversely, this analysis also highlights that the PKIs associated with elevated reporting odds ratios in Figure 2 are detected under conservative conditions, and that these effects relative to a healthy population are likely greater.

### ARRHYTHMIAS BEYOND ATRIAL FIBRILLATION ASSOCIATED WITH PKIs

In the primary text, we describe seven protein kinase inhibitors (PKIs) with significantly increased reporting odds ratios for atrial fibrillation (AF). We hypothesized that the cardiotoxicity of PKIs could lead to electrophysiological diseases other than atrial fibrillation, and accordingly we assessed whether reporting for other types of arrhythmia were also enriched within the adverse event reports for the PKIs. To do so we assessed the following: For each MedDRA Preferred Term denoting a specific arrhythmia classification, the reporting for this term for the seven AF-associated PKIs combined was compared against the reporting within the general FAERS database using χ^2^ disproportionality testing. This was done for each of the 41 preferred terms that exist within the reporting for the seven PKIs. The χ^2^-test was corrected for multiple hypothesis testing using a Bonferroni-correction (n=41). Using the described method, 20 Preferred Terms were identified to be enriched in the PKI population. From this analysis it can be seen that diverse arrhythmia classifications distributed amongst the four MedDRA High Level Terms under Cardiac Arrhythmia are significantly elevated (Supplemental Figure 6). This motivated us to evaluate all PKIs for elevated reporting of all forms of arrhythmia as presented in the primary text.

### AUTHOR CONTRIBUTIONS

JZY set up the logistic regression model and performed analyses shown in primary figures and supplementary figures S2, S4-S7. Analyses were performed with essential inputs from RWM. JZY and FBH set up data sources and vetting. RWM conceived analyses and treatment of comorbidity confounding effects, drug-drug interactions and analyses evaluating apparent protective effects. RWM performed analyses shown in Fig. 1D-E, S1, and S3 with FBH providing essential inputs. FBH and AL conceived the project. AL guided and supervised the project. All authors critically evaluated results and wrote the manuscript.

### REFERENCES

[1] A. Lundby *et al.*, “Oncogenic Mutations Rewire Signaling Pathways by Switching Protein Recruitment to Phosphotyrosine Sites,” *Cell*, vol. 179, no. 2, pp. 543-560.e26, 2019, doi: 10.1016/j.cell.2019.09.008.

[2] F. Carles, S. Bourg, C. Meyer, and P. Bonnet, “PKIDB: A curated, annotated and updated database of protein kinase inhibitors in clinical trials,” *Molecules*, vol. 23, no. 4, pp. 1–18, 2018, doi: 10.3390/molecules23040908.

[3] U. F. & D. Administration, “FDA Adverse Event Reporting System (FAERS): Latest Quarterly Data Files.” https://www.fda.gov/drugs/questions-and-answers-fdas-adverse-event-reporting-system-faers/fda-adverse-event-reporting-system-faers-latest-quarterly-data-files (accessed Oct. 01, 2019).

[4] G. Van Rossum and F. L. Drake Jr, *Python reference manual*. Centrum voor Wiskunde en Informatica Amsterdam, 1995.

[5] W. T. O’Neal *et al.*, “Relation between cancer and atrial fibrillation (from the reasons for geographic and racial differences in stroke study),” *Am. J. Cardiol.*, vol. 115, no. 8, pp. 1090–1094, 2015, doi: 10.1016/j.amjcard.2015.01.540.

[6] J. Alexandre *et al.*, “Identification of anticancer drugs associated with atrial fibrillation - analysis of the WHO pharmacovigilance database,” *Eur. Hear. J. - Cardiovasc. Pharmacother.*, 2020, doi: 10.1093/ehjcvp/pvaa037.

[7] V. Buza, B. Rajagopalan, and A. B. Curtis, “Cancer Treatment-Induced Arrhythmias: Focus on Chemotherapy and Targeted Therapies,” *Circ. Arrhythmia Electrophysiol.*, vol. 10, no. 8, pp. 1–12, 2017, doi: 10.1161/CIRCEP.117.005443.

[8] J. J. Moslehi and M. Deininger, “Tyrosine kinase inhibitor-associated cardiovascular toxicity in chronic myeloid leukemia,” *J. Clin. Oncol.*, vol. 33, no. 35, pp. 4210–4218, 2015, doi: 10.1200/JCO.2015.62.4718.

[9] L. M. Chihara and T. C. Hesterberg, *Mathematical Statistics with Resampling and R*. 2018.


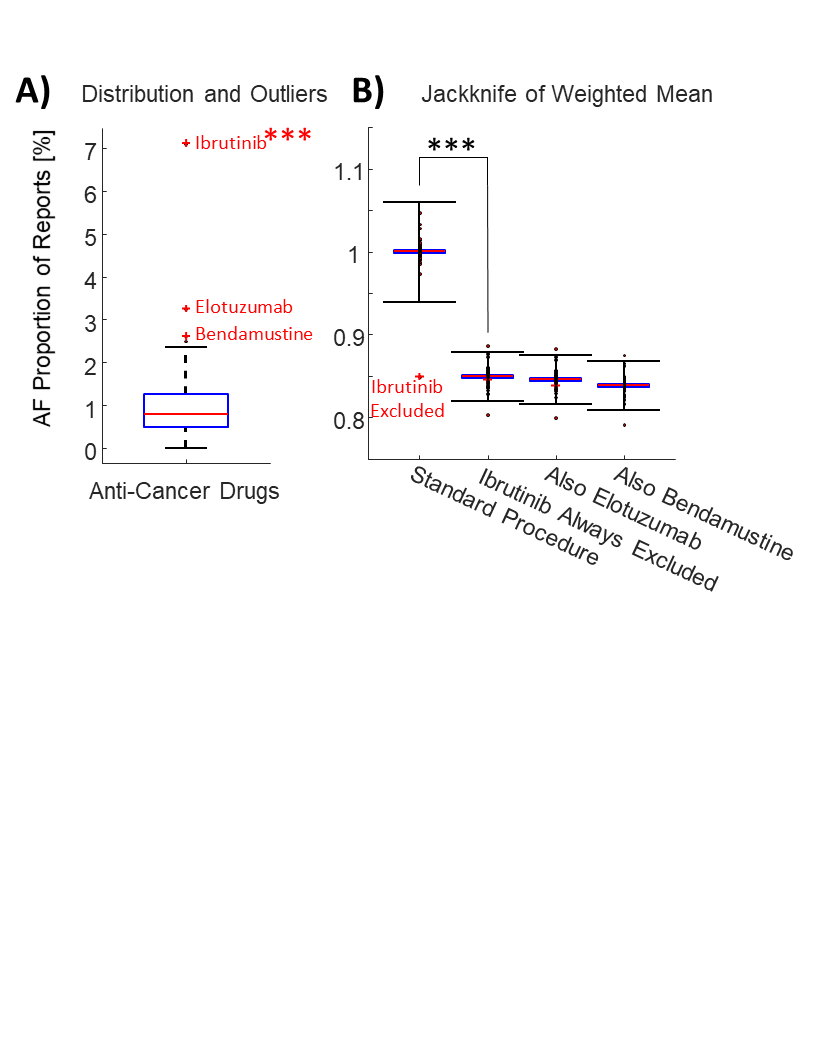


**Supplemental Figure 1** – **Ibrutinib is an extreme outlier skewing the population of reports with anti-cancer drugs**. A) Distribution boxplot of the proportion of reports where atrial fibrillation is the adverse event for each anti-cancer drug; whiskers include all compounds within 1.5x the interquartile range. Ibrutinib is an extreme outlier, more than 7x the interquartile range above Q3 (* p = 1e-10 by Grubb’s test). Less extreme outliers are also noted. B) Boxplots of jackknife-estimates of the weighted-mean of the distribution in panel A; whiskers indicate the Bonferroni-corrected confidence interval. The contribution of each drug to the mean is weighted by the number of reports regarding that drug. This weighted-mean was calculated repeatedly, each time excluding one compound (standard jackknife procedure). The weighted mean that excludes Ibrutinib is an outlier from the rest of the distribution. Repeating the jackknife procedure while systematically excluding Ibrutinib results in a significantly shifted distribution (** p = 2e-14 by paired t-test). In contrast, additional exclusion of the other outlier compounds does not produce further significant shifts of the jackknife distributions.


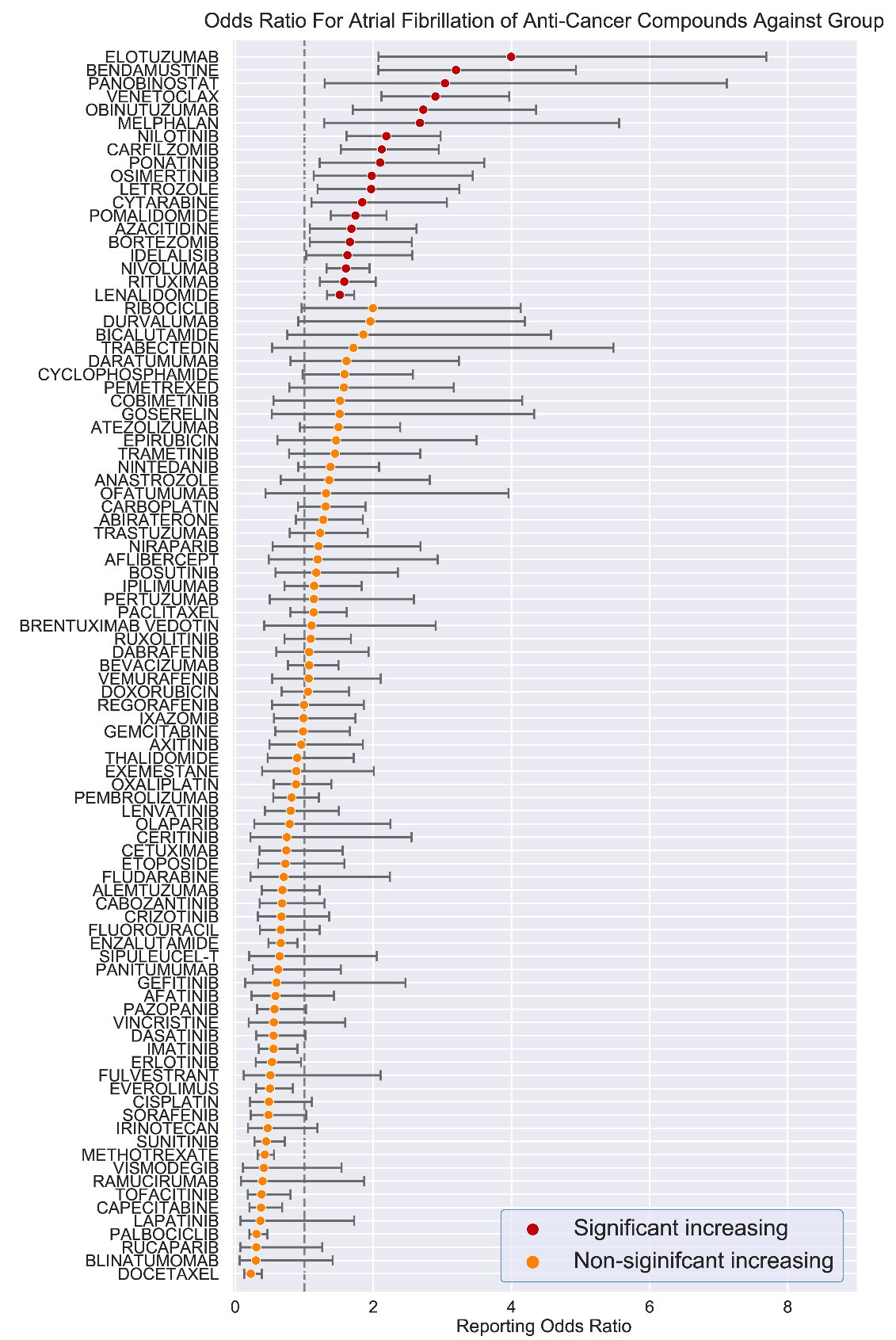


**Supplemental Figure 2** – **Several anti-cancer drugs have elevated atrial fibrillation reporting compared to the combined group of anti-cancer drugs**. Atrial fibrillation reporting odds ratios for each anti-cancer drug individually compared to the entire group combined. Of 178 compounds, only those compounds with at least 500 total reports after vetting were included in the analysis (110 compounds), and only those compounds with at least 5 AF reports are plotted (93 compounds). Odds ratios are shown with Bonferroni-corrected confidence intervals. Compounds with significantly increased reporting odds ratio are shown in red. These significantly elevated compounds were excluded from the anti-cancer drug comparator group for the analysis of PKIs compared to cancer patient reporting.

**
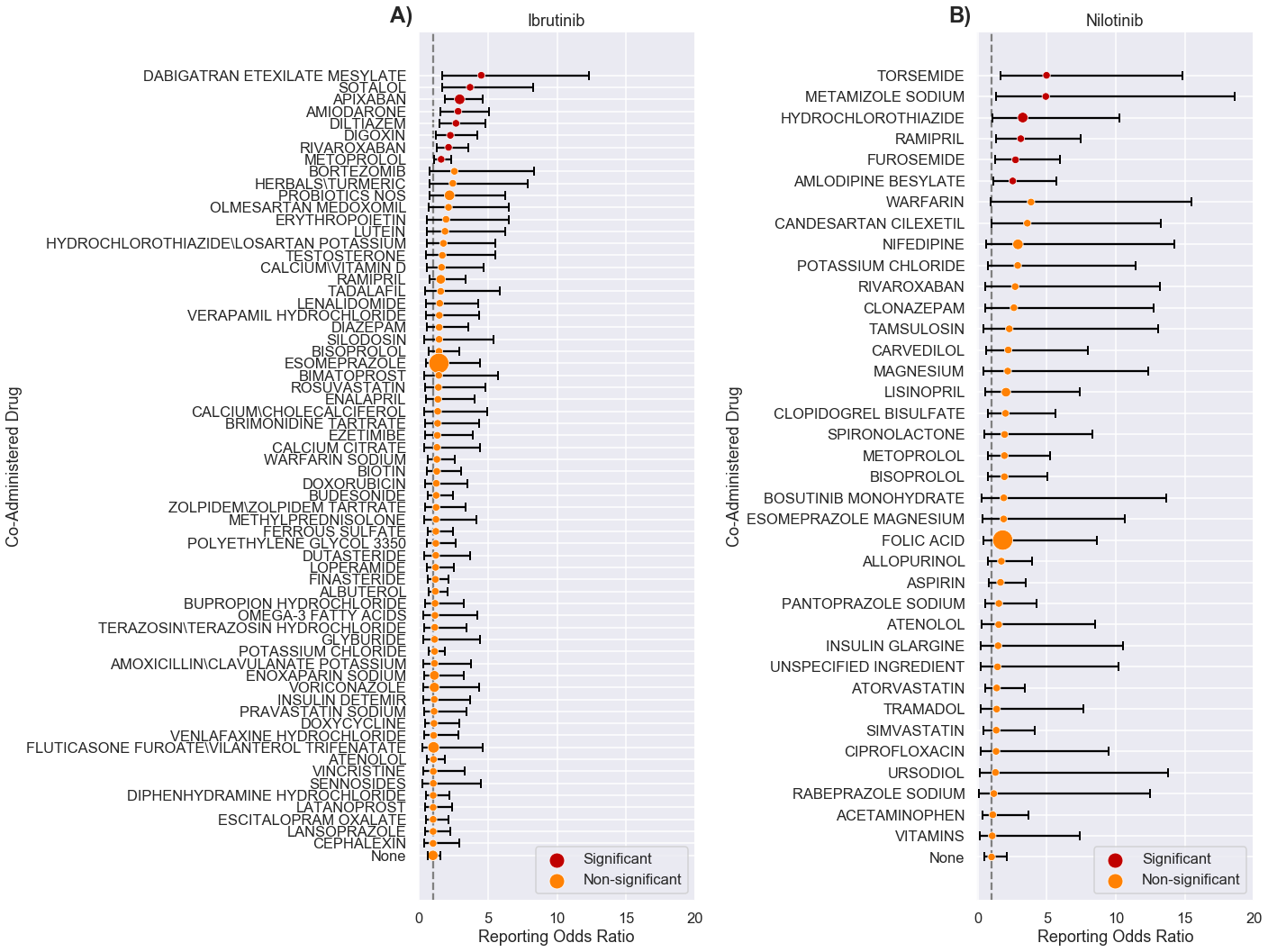
**

**Supplemental Figure 3** – **Elevated AF reporting associated with drugs co-administered with pro-arrhythmic PKIs**. All seven PKIs found to have significantly elevated reporting for atrial fibrillation were assessed by χ^2^ analysis for significant association with co-administration of another drug to detect any drug-drug interactions leading to disease. Only two PKIs had significantly elevated reporting for drugs co-administered with the PKI as compared to the PKI administered alone, (**A**) Ibrutnib and (**B**) Nilotinib. Only co-administered drugs with a Reporting Odds Ratio greater than the PKI alone (‘None’, ROR=1 by definition) are shown with Bonferroni-corrected confidence intervals for multiple testing of all co-administered drugs. Marker size is proportional to total number of reports containing this drug combination.

**Supplemental Figure 4** – **Estimation of adjusted reference population disease reporting rates**. (**A**) Distribution of drug-specific atrial fibrillation reporting rates, where the number of reports regarding atrial fibrillation as a percentage of all adverse event reports is calculated for each non-PKI compound individually. The distribution is heavily skewed with scattered discrete tail values extending up to 13.9%. Annotations indicate the pooled reporting rate present in the conservative analysis of the main text for reference (orange), which is equivalent to the weighted mean rate where the mean calculation is adjusted for the number of reports per drug, and the weighted median (yellow), a more robust measure of central tendency for a skewed distribution. (**B**) As the weighted median is a summary statistic of the distribution, bootstrap methods were used to define the uncertainty interval for this metric. The weighted median was calculated from 100,000 derivative populations, with the distribution of these weighted medians shown. Annotations indicate the pooled reporting rate of the conservative analysis (orange) for reference, as well as the mean weighted-median and the multiple-testing corrected confidence interval for this metric (yellow).


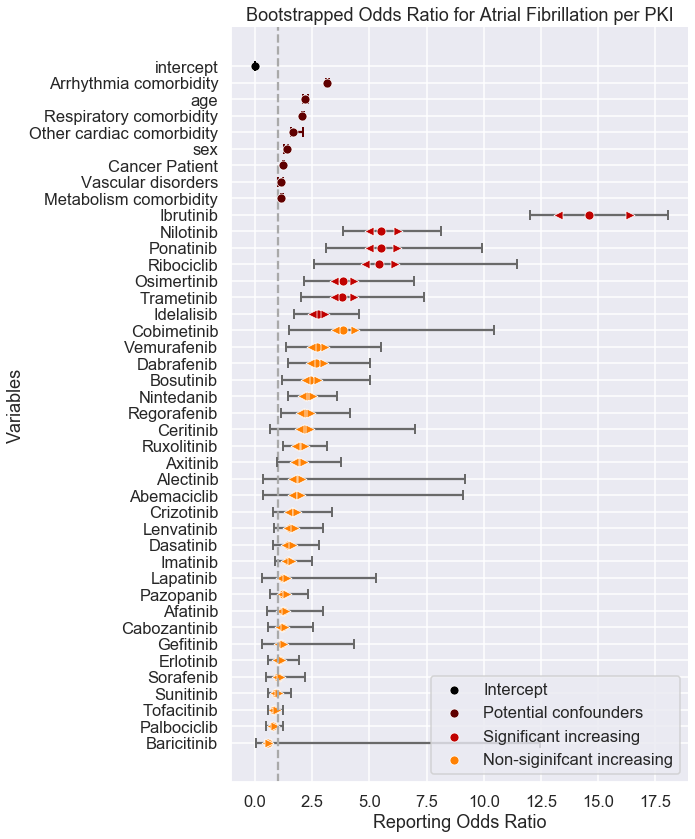


**Supplemental Figure 5** – **Adjusted reference population odds ratios for protein kinase inhibitor effect with regards to development of atrial fibrillation**. Logistic regression analysis was performed after adjusting the reference population to match the estimated background reporting rate after reducing the influence of pro-arrhythmic compounds. Odds ratios presuming the reference population has an atrial fibrillation rate that is the mean rate from the bootstrap distribution of weighted median drug-specific reporting rate (0.36%) is shown with a closed circle. The inner intervals (between triangles) reflect the uncertainty surrounding this background rate estimation, and are constructed by presuming a reference population with a bootstrap confidence interval tail-value rate. Outer confidence intervals are compounded upon the inner CI using the standard errors of the logistic regression parameters. With this analysis, none of the protein kinase inhibitors appear to be protective against atrial fibrillation.


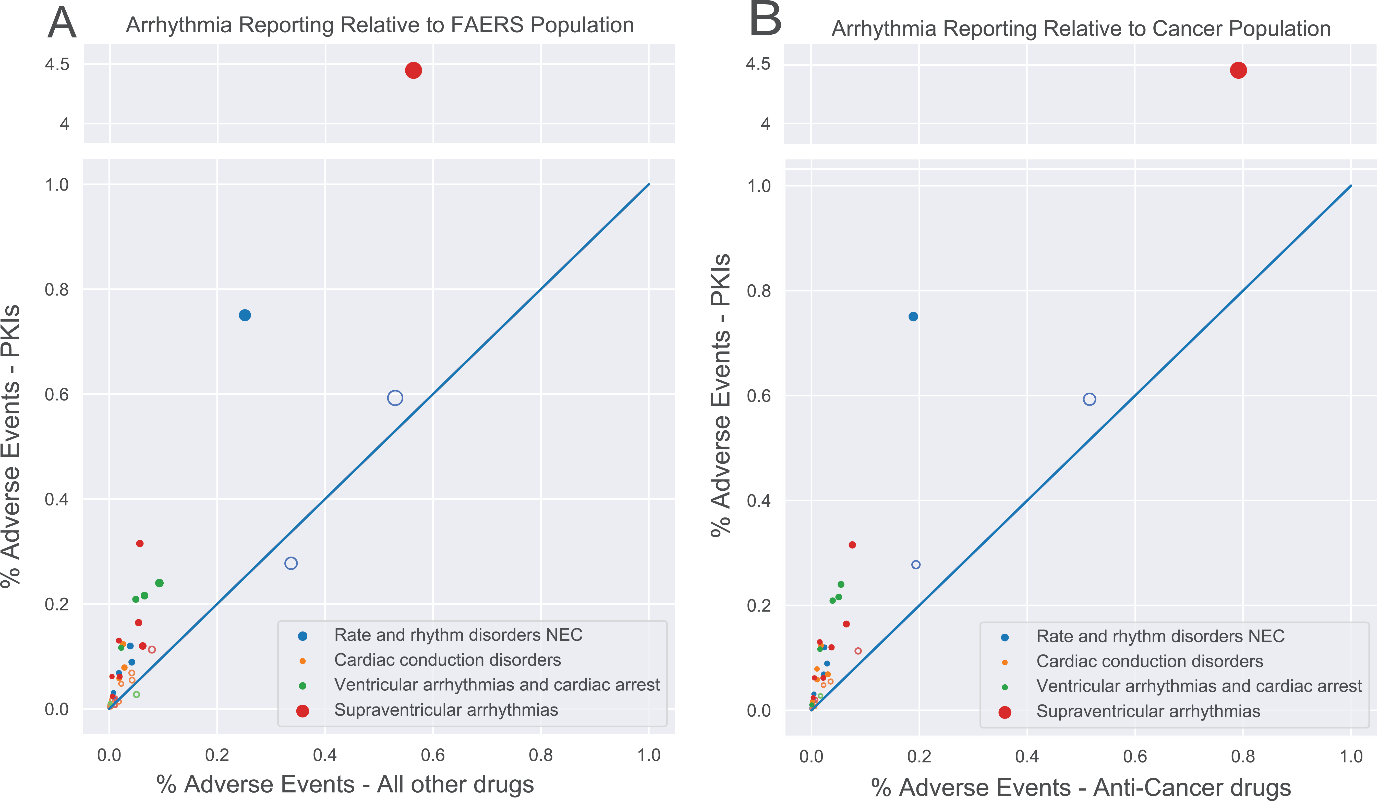


**Supplemental Figure 6** – **Reporting for diverse classifications of arrhythmia are enriched within reports for the atrial fibrillation associated PKIs**. Proportion of adverse events for specific forms of arrhythmia for the seven atrial fibrillation associated PKIs compared to these proportions in the A) FAERS database in general or B) for the collection of reports associated with cancer patients. Filled circles are significantly disproportionate to the comparison group, while empty circles are not. Data point color indicates which MedDRA High Level Term the arrhythmia belongs to, while size is proportional to number of reports. The line indicates unity. Significantly enriched arrhythmias span the MedDRA High Level Terms (in descending % Adverse events – PKIs (y-axis): “'Atrial fibrillation', 'Arrhythmia', 'Atrial flutter', 'Ventricular tachycardia', 'Ventricular fibrillation', 'Ventricular extrasystoles', 'Supraventricular tachycardia', 'Supraventricular extrasystoles', 'Bundle branch block right', 'Cardiac flutter', 'Ventricular arrhythmia', 'Extrasystoles', 'Atrioventricular block first degree', 'Tachyarrhythmia', 'Arrhythmia supraventricular', 'Atrioventricular block second degree', 'Bundle branch block left', 'Sinus node dysfunction', 'Cardiac fibrillation', 'Sinus arrhythmia'). Note that no preferred term was underrepresented in the protein kinase inhibitor group.


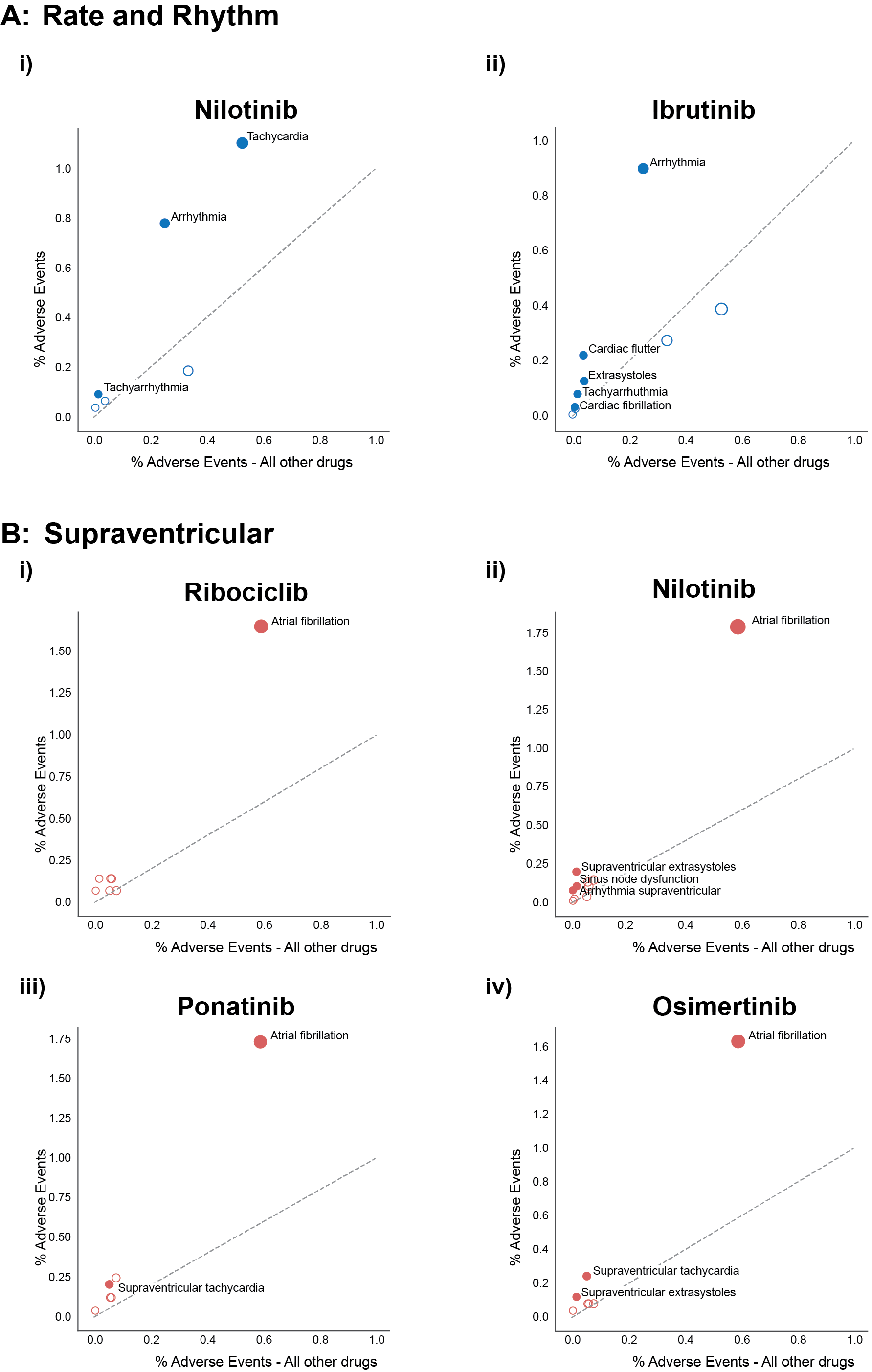


**Supplemental Figure 7** – **Additional post-hoc analyses of Preferred Term support for PKI associations with elevated arrhythmia reporting**. For each compound associated with elevated High Level Term arrhythmia reporting, a post-hoc analysis was performed to determine which underlying specific arrhythmia Preferred Terms had enriched reporting for the specific compound as compared to the general FAERS database in order to assess for recurrent etiological themes. Significantly enriched terms are labelled and shown with filled-in markers, the size of which is proportional to total number of reports for this form of arrhythmia for this drug. (A) Rate and Rhythm Disorders associations for Nilotinib (i) and Ibrutinib (ii) signals were primarily supported by less specific Arrhythmia and Tachyarrhythmia related terms. (B) Supraventricular Arrhythmias associations for Ribociclib (i), Nilotinib (ii), Ponatinib (iii), and Osimertinib (iv) are primarily supported by reporting of Atrial Fibrillation.
